# The aromatase inhibitor letrozole and inhibitors of insulin-like growth factor I receptor synergistically induce apoptosis in *in vitro *models of estrogen-dependent breast cancer

**DOI:** 10.1186/bcr2113

**Published:** 2008-07-08

**Authors:** Joanna Lisztwan, Astrid Pornon, Bin Chen, Shiuan Chen, Dean B Evans

**Affiliations:** 1 Novartis Institutes of BioMedical Research Basel, Oncology Research, Klybeckstrasse 141, CH-4057, Basel, Switzerland; 2 Department of Surgical Research, Beckman Research Institute of the City of Hope, 1500 East Duarte Road, Duarte, CA, 91010, USA

## Abstract

**Introduction:**

Endocrine-dependent, estrogen receptor positive breast cancer cells proliferate in response to estrogens, synthesized by the cytochrome p450 aromatase enzyme. Letrozole is a potent nonsteroidal aromatase inhibitor that is registered for the treatment of postmenopausal women with advanced metastatic breast cancers and in the neoadjuvant, early, and extended adjuvant indications. Because crosstalk exists between estrogen receptor and insulin-like growth factor I receptor (IGF-IR), the effect of combining a selective IGF-IR inhibitor (NVP-AEW541) with letrozole was assessed in two independent *in vitro *models of estrogen-dependent breast cancer.

**Methods:**

MCF7 and T47D cells stably expressing aromatase (MCF7/Aro and T47D/Aro) were used as *in vitro *models of aromatase-driven breast cancer. The role of the IGF-IR pathway in breast cancer cells stimulated only by 17β-estradiol or androstenedione was assessed by proliferation assays. The combination of letrozole and NVP-AEW541 was assessed for synergy in inhibiting cell proliferation using Chou-Talalay derived equations. Finally, combination or single agent effects on proliferation and apoptosis were assessed using proliferation assays, flow cytometry, and immunoblotting.

**Results:**

Both MCF7 and T47D cells, as well as MCF7/Aro and T47D/Aro, exhibited sensitivity to inhibition of 17β-estradiol dependent proliferation by NVP-AEW541. Letrozole combined with NVP-AEW541 synergistically inhibited androstenedione-dependent proliferation in aromatase-expressing cells with combination index values of 0.6 or less. Synergistic combination effects correlated with higher levels of apoptosis as compared with cells treated with the single agent alone. Treatment with either agent also appeared to inhibit IGF-IR signalling via phosphoinositide 3-kinase. Notably, IGF-IR inhibition had limited effect on estrogen-dependent proliferation in the cell lines, but was clearly required for survival, suggesting that the combination of letrozole and IGF-IR inhibition sensitizes cells to apoptosis.

**Conclusion:**

Inhibition of the IGF-IR pathway and aromatase was synergistic in two independent estrogen-dependent *in vitro *models of breast cancer. Moreover, synergism of NVP-AEW541 and letrozole correlated with induction of apoptosis, but not cell cycle arrest, in the cell lines tested. Combination of IGF-IR inhibitors and letrozole may hold promise for the treatment of patients with estrogen-dependent breast cancers.

## Introduction

Breast cancer is the leading cause of death among women in industrialized countries. Approximately two-thirds of breast cancers and breast cancer derived cells exhibit hormone-dependent growth, primarily involving estrogen. Different hormonal therapy approaches that are currently in use or in clinical development for patients with breast cancer prevent either estrogen synthesis or estrogen binding to nuclear estrogen receptors (ERs), thereby downregulating ER-mediated cell proliferation. Letrozole is a potent nonsteroidal aromatase inhibitor, which is an effective treatment for postmenopausal women with advanced breast cancer and in the neoadjuvant, early, and extended adjuvant indications [1-7]. Letrozole acts through reversible binding to the aromatase-cytochrome P450 heme component, thereby blocking the conversion of testosterone and androstenedione (Δ4A) into estrone and 17β-estradiol (E_2_), respectively. A series of studies have shown the effectiveness of letrozole in blocking proliferation of aromatase-expressing, ER-positive tumor cells and its benefits over tamoxifen (for review [2]). Nevertheless, patients may eventually develop resistance through upregulation of growth factor receptor pathways, indicating that a combination therapy approach would be more beneficial in terms of prolonging patient response to letrozole [8].

The insulin-like growth factor I receptor (IGF-IR) is a transmembrane receptor tyrosine kinase that is activated by its ligands, insulin-like growth factor (IGF)-I and IGF-II, as well as to some extent insulin [9]. Activation of tyrosine kinase activity upon ligand binding results in trans-subunit auto-phosphorylation of the receptor and stimulation of signaling cascades, including the mitogen-activated protein kinase (MAPK) and phosphoinositide 3-kinase (PI3K) pathways. IGF-IR signaling has been reported to promote proliferation, growth, survival, transformation, metastasis, and angiogenesis. Moreover, high levels of IGF-IR and/or its activating ligands IGF-I and IGF-II have been associated with various types of cancers [10,11]. In keeping with these observations, targeted over-expression of IGF-IR in various tissues (including mammary gland, pancreatic islets, and basal epidermis) results in tumor development and, in some cases, formation of metastases in mice [12-15]. Conversely, inhibition of tumor growth in different mouse models was observed using antibodies that are capable of blocking IGF-IR auto-phosphorylation and signaling, or selective small-molecular-weight inhibitors against the receptor [16,17].

NVP-AEW541 (referred to subsequently in this report as AEW541) was characterized as an orally bioavailable, selective IGF-IR inhibitor for the targeted treatment of various tumor types that are dependent on IGF-IR-mediated signaling for proliferation/survival [18]. Notably, although AEW541 exhibits antiproliferative/proapoptotic responses *in vitro *and antitumor activity *in vivo *as a single agent, it is believed that IGF-I signaling inhibition could be most effective in combination with other therapies [19]. For example, AEW541 enhanced suppression of Ewing's sarcoma xenograft growth when combined with vincristine [20].

Existing evidence indicates that ER and IGF-IR exhibit bidirectional regulation *in vitro *and *in vivo *[16,21,22]. *In vitro*, estrogen and IGF-I were shown to act synergistically to induce S-phase entry of MCF7 breast cancer cells [23,24]. In the breast, crosstalk between the two pathways plays a key role in the development of normal mammary gland as well as breast carcinoma [25]. Moreover, the expressions of ER and IGF-I family members have prognostic significance [26-32], and there is a positive correlation between IGF-I concentration and risk for cancer in premenopausal women [33]. Considering that a strong link exists between both receptors and their involvement in breast cancer progression, a combination of ER and IGF-I pathway inhibitors represents a rational therapeutic strategy. We determined to begin examining this issue using *in vitro *models of estrogen-dependent breast cancer.

## Materials and methods

### Cell culture

MCF7/Aro, MCF7 3(1), T47D/Aro and T47D, human breast carcinoma cells stably transfected with the aromatase or empty control expression vectors, respectively, carrying the neomycin (G418) resistance gene, were generated and characterized by Sun and coworkers [34]. MCF-7/Aro is a very stable cell line and aromatase expression remains in the absence of G418, whereas T47D/Aro requires G418 during culture. All cell lines are dependent on E_2 _for cell proliferation. Cells were therefore maintained in minimum essential medium with Earle's balanced salts (MEM-EBS) supplemented with 10% fetal calf serum (FCS), 2 mmol/l L-glutamine, 1 mmol/l sodium pyruvate, nonessential amino acids, penicillin/streptomycin, and 0.5 mg/ml G418 (BioConcept, Basel, Switzerland). Estrogen-induced cell proliferation assays were performed with steroid-depleted medium, in which MEM-EBS and FCS were replaced with MEM-EBS without phenol red and charcoal-stripped FCS (Hyclone, Logan, UT, USA). Proliferation was induced with Δ4A or E_2 _(Sigma-Aldrich, St Louis, MO, USA), which were dissolved in ethanol as stock solutions of 10 mmol/l and 1 mmol/l, respectively. NVP-AEW541 and letrozole were synthesized in the laboratories of Novartis Institutes for Biomedical Research (Basel, Switzerland) and were dissolved in dimethyl sulfoxide (DMSO; 10 mmol/l) and ethanol (1 mmol/l) as stock solutions.

### *In vitro *proliferation assay

MCF7 3(1) and MCF7/Aro cells at a confluency of 30% to 40% were steroid-deprived for 3 days, trypsinized with trypsin lacking phenol red (Sigma-Aldrich), and seeded in six-well plates (10^5 ^MCF7/Aro cells per well and 1.2 × 10^5 ^MCF7 3(1) cells per well). Two days later, steroid-deprived media containing 1 nmol/l E_2 _or 10 nmol/l Δ4A was added to the cells to induce proliferation. Vehicle control or compound was added simultaneously and left for 6 days on the cells. Media, E_2_, or Δ4A, and compound were renewed every 48 hours. Each condition was tested in triplicate. After treatment, cells were fixed by addition of 20% glutaraldehyde, stained by addition of 0.05% methylene blue, washed with water to remove excess methylene blue, and 3% HCl was added to each well to dissolve the methylene blue stain. The absorbance was read at 650 nmol/l, and percentage inhibition was calculated using Softmaxpro software (Molecular Devices Corp., Sunnyvale, CA, USA). More specifically, cells treated with either E_2 _or Δ4A in the presence of DMSO were considered to represent 100% proliferation, and subsequently compound-treated cells were compared with these standards.

T47D and T47D/Aro cells were similarly steroid-deprived for 3 days and then seeded in 96-well plates (6,000 cells per well). Steroid-deprived cells were treated with hormone and compound every other day for 6 days, as described above. Each condition was tested in triplicate. Proliferation assay was carried out using a CellTiter 96^® ^AQueous One Solution Cell Proliferation Assay (Promega, Madison, WI, USA), and percentage inhibition was calculated as described above.

### Statistical analysis

Combination studies were performed at a ratio of 4.85:1 (letrozole:AEW541) in MCF7/Aro cells, and 20:1 (AEW541:letrozole) in T47D/Aro cells. Each condition was tested in triplicate (single agent and combination) and the average percentage inhibition of cell growth in response to specific compound concentrations was entered into the CalcuSyn program for dose-effect analysis (Biosoft, Cambridge, UK). Median effect and combination index were calculated in accordance with the Chou-Talalay derived equations [35] and data are represented as nonexclusive Monte Carlo values. Combinations were performed at a constant ratio (derived from the calculated average 50% inhibitory concentration [IC_50_] values).

### Flow cytometry analysis

To analyze the cell cycle profile, treated MCF7/Aro or T47D/Aro cells were fixed overnight with 70% EtOH at -20°C and stained with propidium iodide buffer (38 mmol/l sodium citrate [pH 7.5], 69 μmol/l propidium iodide, and 120 μg/ml RNase A). To analyze the numbers of apoptotic cells, treated MCF7/Aro cells were processed for Annexin V/7-AAD (Becton Dickinson, San Diego, CA, USA) staining, inn accordance with the manufacturer's instructions. Cell cycle distribution and percentage of apoptotic cells was analyzed with a Becton Dickinson FACSCalibur flow cytometer. More specifically, cells were gated for DNA staining (FLH-3, 7-AAD) and apoptosis (FLH-1, Annexin V), whereby apoptotic cells are represented in the two right-hand quadrants of the FACS image (high FLH-1 staining).

### Western blotting

To analyze levels of signaling proteins, the treated MCF7/Aro cells were processed for Western blotting with the following antibodies: anti-IGF-IRβ (C-20; Santa Cruz Biotechnology, Santa Cruz, CA, USA), anti-IRS-1, anti-PKBα (Upstate, Charlottesville, VA, USA), anti-phospho-IRS-1 (pY612; Invitrogen, Carlsbad, CA, USA), anti-ERα (DakoCytomation, Glostrup, Denmark), anti-phospho-PKB (Ser473), anti-p44/42 MAPK, anti-phospho-p44/42 MAPK (Thr202Tyr204), anti-human caspase 9, anti-PARP (Cell Signaling Technology Inc., Beverly, MA, USA), and anti-β-actin (Abcam, Cambridge, UK).

## Results

### Estrogen-dependent proliferation of MCF7/Aro and T47D/Aro cells exhibits sensitivity to IGF-IR inhibition

In order to assess the role played by IGF-IR in E_2_-induced proliferation, we selected two established ER-positive breast cancer cell lines, namely MCF7 and T47D. MCF7 cells are known to be E_2 _dependent as well as very sensitive to the growth inhibitory effects of AEW541 in an IGF-I-dependent survival assay (IC_50 _162 ± 16 nmol/l) and a soft agar anchorage-independent growth assay (IC_50 _105 ± 18 nmol/l) [18]. By steroid-depriving either the parental cells or cells over-expressing aromatase (namely MCF7/Aro and T47D/Aro), we determined whether AEW541 can inhibit their E_2_-dependent proliferation. Table 1 summarizes the dose-dependent inhibitory effect of AEW541 on E_2_-dependent growth of the cell lines in three independent experiments. Notably, in IGF-I-dependent proliferation assays the analysis of MCF7 cells revealed an IC_50 _value of 150 ± 8 nmol/l, pointing to a key role of IGF-IR in mediating estrogen-dependent proliferation in MCF7 cells [22]. T74D cells also exhibited sensitivity to AEW541, albeit at about fourfold higher concentrations as compared with MCF7 cells (IC_50 _values: 150 ± 8 nmol/l versus 544 ± 133 nmol/l).

**Table 1:** Estrogen-dependent proliferation of breast cancer cell lines is dependent on IGF-IR

Cell line	Compound	E_2 _IC_50 _(nmol/l)	Δ4A IC_20_^a ^(nmol/l)	Δ4A IC_50 _(nmol/l)	Δ4A IC_80_^a ^(nmol/l)
MCF7 3(1)	AEW541	150 ± 8	NA	NA	NA
MCF7/Aro	AEW541	91 ± 37	66 ± 15	130 ± 16	280 ± 36
	Letrozole	NA	150 ± 8	444 ± 89	1,160 ± 372

T47D	AEW541	544 ± 133	NA	NA	NA
T47D/Aro	AEW541	612 ± 124	733 ± 422	4,474 ± 2,613	31,272 ± 2,300
	Letrozole	NA	9 ± 7	67 ± 29	2171 ± 502

Steroid-deprived MCF7, MCF7/Aro, T47D, and T47D/Aro cells were treated with either 17β-estradiol (E_2_) or androstenedione (Δ4A) in the presence of increasing concentrations of letrozole or AEW541 for 6 days. Percentage inhibition of proliferation was determined as described in the text. The average inhibitory concentrations at 20%, 50%, and 80% (IC_20_, IC_50_, and IC_80_, respectively) ± standard deviation was calculated for MCF7 cell lines. ^a^IC_25 _and IC_75 _values were calculated for T47D cell lines. IGF-IR, insulin-like growth factor I receptor; NA, not applicable.

### Combination of letrozole and IGF-IR inhibition is synergistic for inhibition of estrogen-dependent proliferation

**Figure 1:**
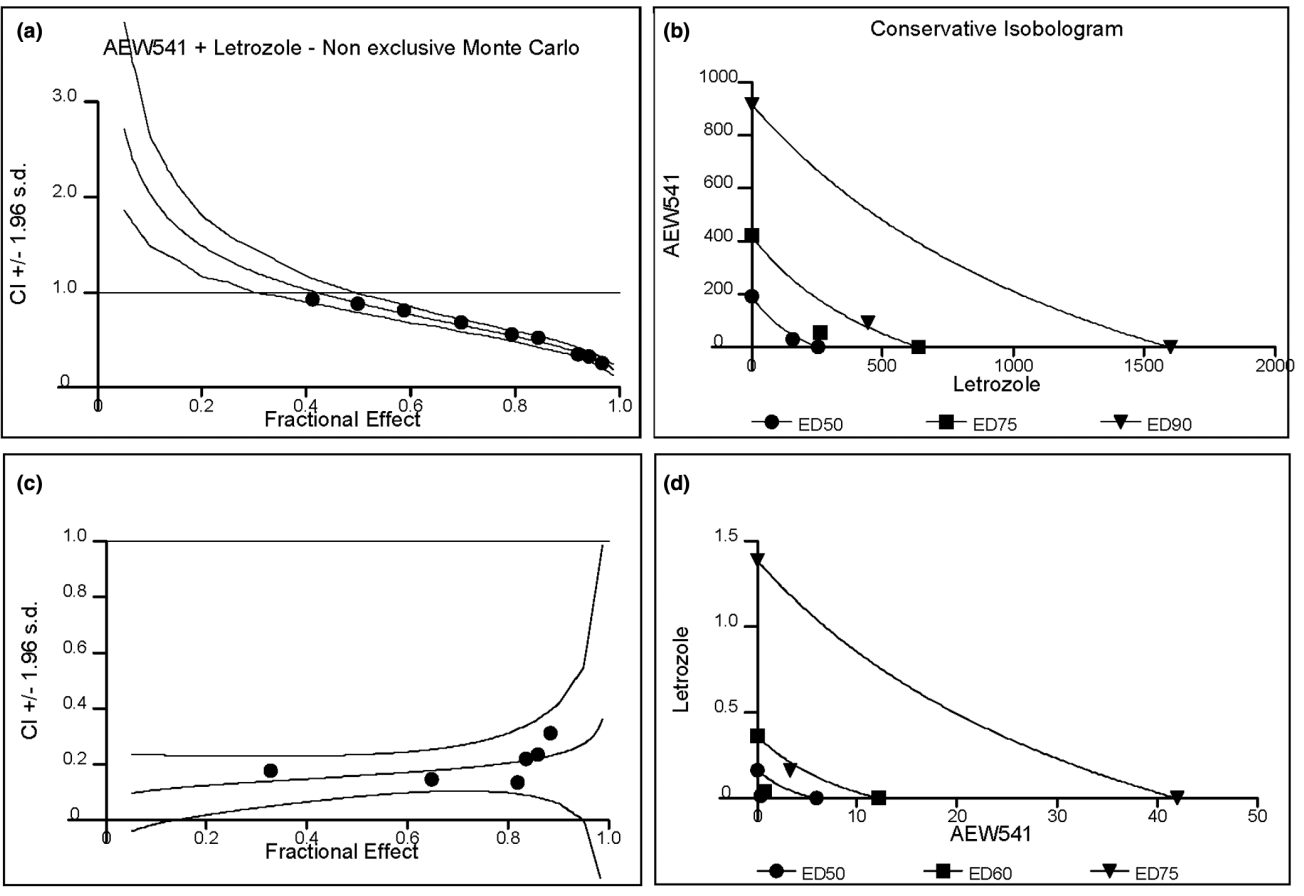
Combination of AEW541 and letrozole is synergistic in inhibiting androstenedione-dependent proliferation. Steroid-deprived MCF7/Aro (upper panels) and T47D/Aro (lower panels) cells were treated with androstenedione (Δ4A) in the presence of increasing concentrations of letrozole and/or AEW541 for 6 days. Percentage inhibition of proliferation was determined (as described in the text), and combination index (CI) values of the two agents plotted, according to a **(a,c) **nonexclusive Monte Carlo extrapolation. **(b,d) **Similarly, a conservative isobologram was plotted. ED, effective dose; s.d., standard deviation.

In order to establish the appropriate experimental conditions in which to monitor the effects of letrozole as a single agent or in combination, it was necessary to replace E_2 _with Δ4A, an androgen that is processed by aromatase. By titrating AEW541 or letrozole onto MCF7/Aro or T47D/Aro cells in the presence of Δ4A, we observed a dose-dependent inhibition of cell growth by both AEW541 and letrozole in three independent experiments (Table 1). Using IC_20_, IC_50_, and IC_80 _values, we determined an appropriate concentration range for both letrozole and AEW541 in the combination study. Each experiment required titration of AEW541 and letrozole as single agents as well as in combination at a fixed ratio. Percentage inhibition of cell proliferation for single agent concentrations and combination concentrations were entered into CalcuSyn and the median effect and combination index calculated according to the Chou-Talalay derived equations [35] (Figure 1, Table 2, and Additional file 1).

**Table 2:** Summary of combination index values for AEW541 and letrozole

Cell line	Fa	Compound	IC_50 _(nmol/l)	DRI	CI
MCF7/Aro	0.5	AEW541	33	5.9	0.888
		Letrozole	158	1.6	
	0.75	AEW541	55	7.7	0.597
		Letrozole	265	2.4	
	0.9	AEW541	92	10.0	0.406
		Letrozole	445	3.6	

T47D/Aro	0.5	AEW541	268	7.9	0.513
		Letrozole	13	3.0	
	0.75	AEW541	3326	10.0^a^	0.284
		Letrozole	166	4.4	

Combination index (CI) values above 1.1 indicate antagonistic, 0.9 to 1.1 additive, 0.7 to 0.9 moderately synergistic, 0.3 to 0.7 synergistic, and under <0.3 strongly synergistic. Note that the effective dose at 50%, 75% and 90% inhibition is decreased when both agents are combined. ^a^DRI is greater than 10 (outside of concentration range). DRI, dose response index; Fa, fraction affected; IC50, 50% inhibitory concentration.

Three independent experiments revealed excellent linear correlation coefficient *r *values (>0.97) and dose-effect fit curves (data not shown). Moreover, the combination index curves (Figure 1a,c) and conservative isobolograms (Figure 1b,d) indicated synergism of activity between letrozole and AEW541 in both cell lines. More specifically, the data indicate that AEW541 and letrozole are synergistic, especially at higher affected fractions (Fa 0.75 and 0.9), for which combination index is 0.6 or less (Table 2). Strikingly, dose response index values for AEW541 were generally higher than for letrozole (ranging from 5.2 to 10 for AEW541 versus 1.1 to 6.5 for letrozole), indicating AEW541 concentrations could be strongly decreased in the presence of letrozole. In summary, these data suggest that a combination of IGF-IR and aromatase inhibition in estrogen-dependent breast cancer cell lines is synergistic in terms of inhibitory activity.

### Combination of AEW541 and letrozole are not synergistic in inducing a cell cycle arrest

In order to understand the mechanisms that underlie the synergistic antiproliferative actions of AEW541 and letrozole, cell cycle profiles were analyzed following addition of these agents. The most reliable concentrations, corresponding to the above calculated Fa 0.75 (T47D/Aro) or 0.9 (MCF-7/Aro; Table 2), were chosen to be used alone or in combination. Note that although concentrations were within an acceptable range for both agents, namely close to the single agent IC_50 _values for both AEW541 and letrozole, they were still suboptimal for achieving IC_90 _(or IC_75_) and therefore allowed detection of synergistic effects. Table 3 summarizes cell cycle profile results obtained in one experiment, although data looked highly comparable over a set of three independent experiments.

**Table 3:** Summary of altered cell cycle distribution in response to treatment with single agent or in combination

Cell line	Compound	Sub-G_1 _(%)	G_1 _phase (%)	S phase (%)	G_2_/M phase (%)	Annexin/7-AAD (%)
MCF7Aro (24 hours)	-Δ4A	2.5	77.7	11.1	9.3	3.5
	+Δ4A	1.6	39.9	41.8	17.5	2.8
	AEW541 (92 nmol/l)	2.2	44.0	39.1	15.1	3.2
	Letrozole (445 nmol/l)	3.1	74.2	12.8	10.7	2.7
	AEW541 + Letrozole	2.4	78.4	10.1	9.5	2.3

T47D/Aro	-Δ4A	0.6	86.5	4.1	8.1	ND
	+Δ4A	0.6	77.9	10.1	10.7	ND
	AEW541 (3.3 μmol/l)	0.3	83.8	6.2	9.0	ND
	Letrozole (165 nmol/l)	0.4	84.2	6.5	8.3	ND
	AEW541 + letrozole	0.4	87.3	3.8	8.1	ND

MCF7/Aro (96 hours)	-Δ4A	6.2	64.8	4.9	24.4	23.7
	+Δ4A	1.8	65.3	13.0	20.4	5.8
	AEW541 (92 nmol/l)	2.6	59.0	14.8	23.9	5.5
	Letrozole (445 nmol/l)	3.3	59.0	14.1	24.0	11.2
	AEW541 + Letrozole	7.3	58.7	12.2	22.4	22.5

T47D/Aro (96 hours)	-Δ4A	3.9	77.9	4.8	11.9	ND
	+Δ4A	0.9	68.2	16.7	13.4	ND
	AEW541 (3.3 μmol/l)	4.4	71.0	12.6	10.7	ND
	Letrozole (165 nmol/l)	1.6	80.8	5.8	10.8	ND
	AEW541 + letrozole	5.0	77.2	4.3	11.7	ND

Steroid-deprived MCF7/Aro and T47D/Aro cells were treated for 24, 48, or 96 hours with either no androstenedione (Δ4A; -Δ4A), Δ4A in the presence of dimethyl sulfoxide (+Δ4A), or Δ4A in the presence of the indicated concentrations of compound. Cells were processed for fluoresence-activated cell sorting (FACS) using propidium iodide and AnnexinV/7-AAD staining, as described in the text. Percentage distribution of cells in each cell cycle phase and total Annexin V stained cells are shown. The percentage value corresponds to upper and lower right quadrants of FACS plots in Figure 4b. ND, not determined.

**Figure 2:**
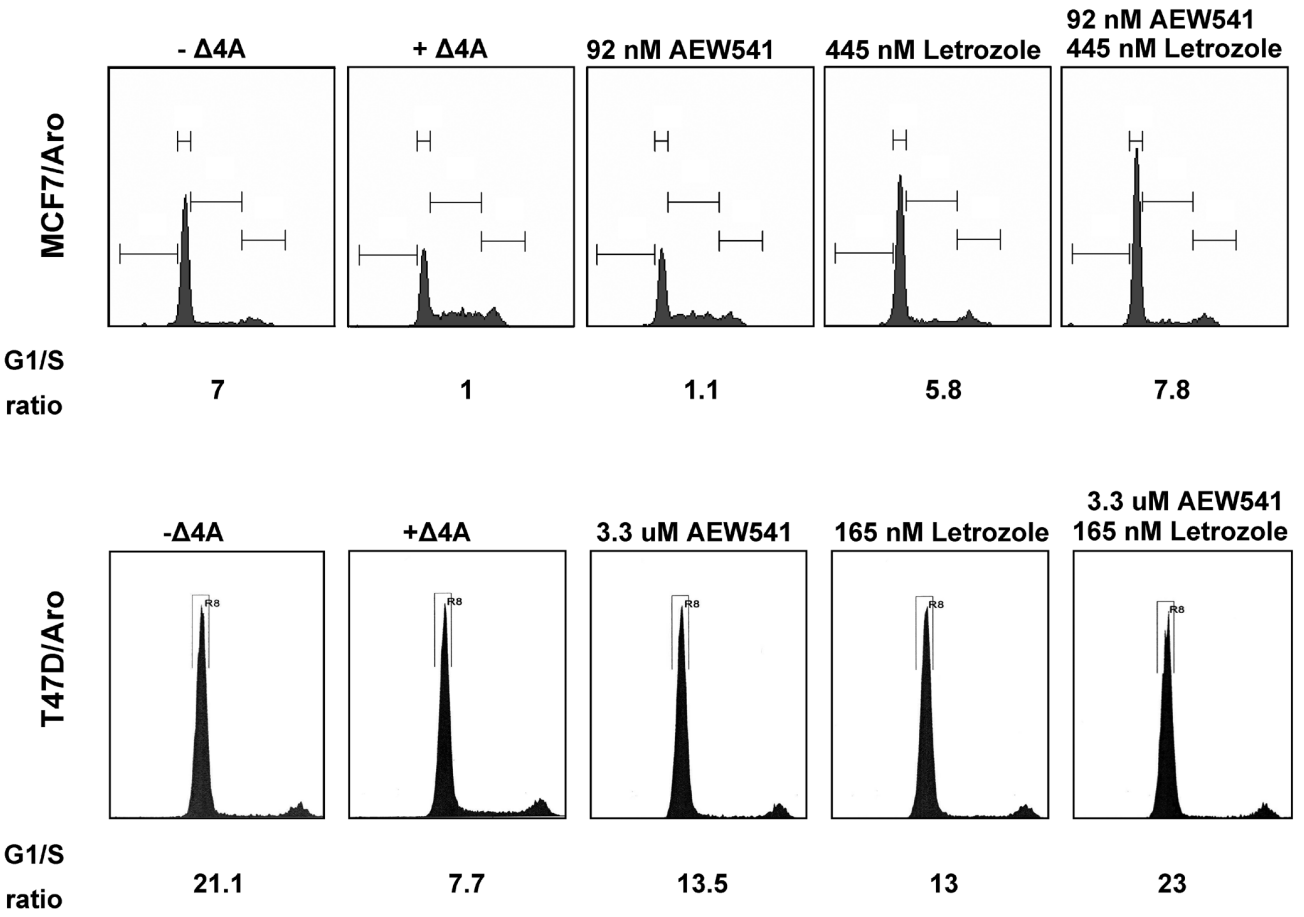
Letrozole, but not AEW541, shows antiproliferative activity at suboptimal concentrations. Steroid-deprived MCF7/Aro and T47D/Aro cells were treated for 24 and 48 hours, respectively, with either no androstenedione (Δ4A; -Δ4A), Δ4A in the presence of dimethyl sulfoxide (DMSO; +Δ4A), or Δ4A in the presence of the indicated concentrations of compound. Cells were processed for fluorescence-activated cell sorting using propidium iodide (as described in the text). G_1_/S ratio was calculated by dividing G_1 _phase values by S phase values (see Table 3).

Normal cell cycle progression in our system was clearly dependent on estrogen, as indicated by an accumulation in G_1 _phase in the absence of Δ4A (Figure 2 and Table 3; components labeled -Δ4A). Moreover, a normal cell cycle distribution was observed in the presence of Δ4A (Figure 2 and Table 3; components labeled +Δ4A). When letrozole was added as a single agent or in combination with AEW541, cell cycle profiles resembled that of cells grown in the absence of Δ4A (Table 3). More specifically, MCF7/Aro cells were 78% in G_1 _without Δ4A, 40% with Δ4A, and 74% and 78% in the presence of Δ4A and letrozole (similar for T47D/Aro, with values of 87%, 78%, 84%, and 87% in G_1 _phase, respectively). While AEW541 alone exhibited almost no effects on the cell cycle profile in MCF7/Aro cells (compare 40% G_1 _versus 44%), T47D/Aro cells accumulated in G_1 _phase to almost as high levels as without Δ4A (compare 78% G_1 _versus 84%). This would suggest a different requirement for IGF-IR signaling in each cell line. Nevertheless, the synergism between the two agents *in vitro *cannot be accounted for by a synergistic induction of cell cycle arrest.

### Estrogen-dependent IGF-IR signaling via PI3K is altered by both letrozole and AEW541

Previous work has established bidirectional regulation of the ER and IGF-IR pathways. For example, ER signaling can lead to an upregulation of IGF-I family members [21,22], and IGF-IR signaling activates ER-mediated gene transcription [36]. We therefore addressed, at the molecular level, the synergistic mechanism of letrozole and AEW541 regarding proliferation and apoptosis of MCF7/Aro cells. This required analysis of two independent time points, namely an early 24-hour time point (for proliferation events) and a late 96-hour time point (for apoptosis events). Data are again presented from one experiment but were reproducible over three independent experiments.

**Figure 3:**
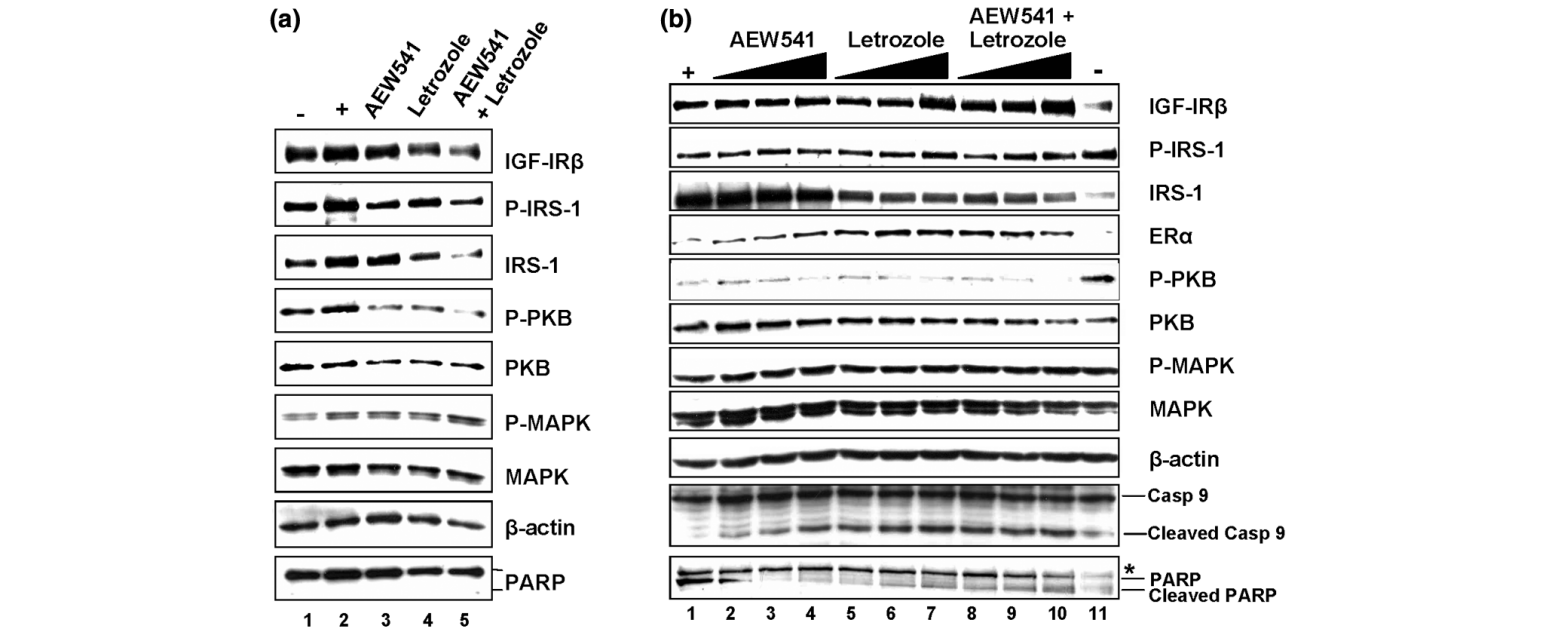
Letrozole and AEW541 both modulate IGF-IR pathway signaling via the PI3K/PKB axis. **(a) **Steroid-deprived MCF7/Aro cells were treated for 24 hours with either no androstenedione (Δ4A; lane 1), Δ4A in the presence of dimethyl sulfoxide (DMSO; lane 2), or Δ4A in the presence of 92 nmol/l AEW541 (lane 3) or 445 nmol/l letrozole (lane 4), or both combined (lane 5). Cells were processed for Western blotting with specific antibodies for the proteins indicated. Note that samples were processed in parallel to fluoresence-activated cell sorting (FACS) data shown in Figure 2. **(b) **Steroid-deprived MCF7/Aro cells were treated for 96 hours with either no Δ4A (lane 11), Δ4A in the presence of DMSO (lane 1), or Δ4A in the presence of 33, 55, and 92 nmol/l AEW541 (lanes 2 to 4), 158, 265 and 445 nmol/l letrozole (lanes 5 to 7), or both respectively combined (lanes 8 to 10). Cells were processed for Western blotting with specific antibodies for the proteins indicated. Note that samples were processed in parallel to FACS data displayed in Figure 4. *Background band. Casp, caspase; IGF-IR, insulin-like growth factor I receptor; IRS, insulin receptor substrate; MAPK, mitogen-activated protein kinase; PARP, poly [ADP-ribose] polymerase; PKB, protein kinase B.

In order to monitor effects on proliferation, cells were stimulated simultaneously with Δ4A and treated for 24 hours with both agents alone or in combination (Figure 3a) before processing for Western blotting. As expected, IGF-IRβ and insulin receptor substrate (IRS)-1 protein levels as well as phosphorylation of IRS-1 increased upon addition of Δ4A (Figure 3a; compare lanes 1 and 2). Letrozole and AEW541 both reverted these effects, and combination of the two exhibited additive inhibition (Figure 3a; compare lane 2 versus lanes 3 to 5). Protein and phospho-protein levels of two independent downstream signaling kinases, namely protein kinase B (PKB)/Akt and MAPK, were also assessed. Strikingly, phospho-MAPK levels appeared to be unaffected by either compound treatment, whereas phospho-PKB/Akt levels decreased in the presence of either agent (Figure 3a; compare lane 2 versus lanes 3 and 4). Moreover, combination of the two compounds was additive in lowering phospho-PKB/Akt levels as well as upstream pathway members IGF-IRβ, IRS-1, and phospho-IRS-1 (Figure 3a; compare lane 2 versus lanes 3, 4 and 5). ERα was not detectable (data not shown). Hence, combination of AEW541 and letrozole appears to inhibit PI3K (but not MAPK) signaling to a greater extent than either agent alone.

Morphologic analysis of cells over a period of 6 days revealed that dead cells began to appear 4 days after compound treatment. Therefore, cells were treated for 96 hours simultaneously with Δ4A and increasing concentrations of either agent alone or in combination, before being processed for Western blotting. Addition of Δ4A led to the upregulation of IGF-IRβ and IRS-1 protein levels (Figure 3b; compare lanes 1 and 11), as also seen at 24 hours (Figure 3a). Confirming previous work [37], letrozole induced a modest upregulation of ERα and IGF-IRβ in the presence of Δ4A after 96 hours, but dramatically decreased levels of IRS-1 (Figure 3b; compare lane 1 versus lanes 5 to 7). Interestingly, even after 4 days of compound treatment, no effect on phospho-protein levels of MAPK or a related family member, p38MAPK, were observed (Figure 3b and Additional file 2). However, phospho-PKB/Akt levels remained inhibited by both AEW541 and letrozole in a concentration-dependent manner (Figure 3b; compare lane 1 versus lanes 2 to 4 and 5 to 7). This inhibition was again additive when both agents were combined for 96 hours (Figure 3b; compare lanes 4 and 7 versus lane 10). Interestingly, culturing cells for 4 days in the absence of Δ4A led to an upregulation of phospho-PKB and, to a lesser degree, phospho-IRS-1 (Figure 3b, lane 11), whereas phospho-MAPK and phospho-p38MAPK levels remained unchanged, once again highlighting the importance of PI3K/PKB/Akt signaling in mediating survival when estrogen signals are absent.

Hence, our data indicate that MCF7/Aro cells lose estrogen survival signals after addition of letrozole, which can most easily be monitored through changes in IRS-1, ER [37], and IGF-IR [38] protein levels, even at suboptimal concentrations. These survival signals appear to be mediated by PI3K/PKB/Akt signaling, which is additively suppressed by addition of both AEW541 and letrozole. Notably, although there were similarities between the molecular events monitored at 24 and 96 hours, clear differences in phospho-PKB and phospho-IRS-1 responses could be observed, emphasizing the need to discriminate between early and late events when studying molecular responses to drug treatment.

### AEW541 and letrozole treatment shows combined proapoptotic potential

**Figure 4:**
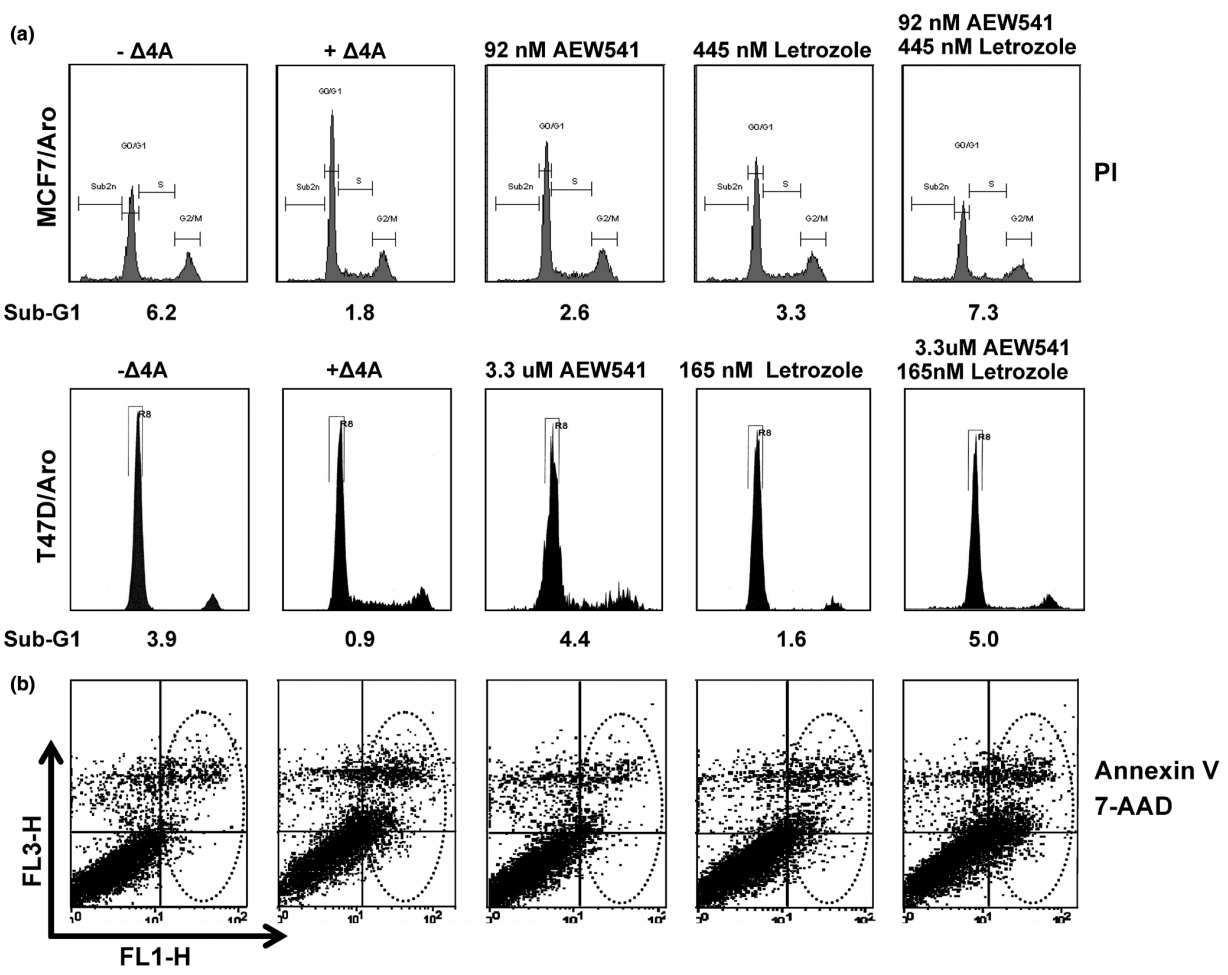
Letrozole and AEW541 exhibit synergistic proapoptotic activity at suboptimal concentrations. **(a) **Steroid-deprived MCF7/Aro and T47D/Aro cells were treated for 96 hours with either no Δ4A (-Δ4A), Δ4A in the presence of dimethyl sulfoxide (DMSO; +Δ4A), or Δ4A in the presence of the indicated concentrations of compound. Cells were processed for fluoresence-activated cell sorting (FACS) using propidium iodide as described. Percentage of cells in sub-G_1 _is indicated. **(b) **Steroid-deprived MCF7/Aro cells were treated for 96 hours with either no Δ4A (-Δ4A), Δ4A in the presence of DMSO (+Δ4A), or Δ4A in the presence of the indicated concentrations of compound. Cells were processed for FACS using 7-AAD/Annexin V staining and apoptotic subpopulations identified as described in the Materials and methods section. Note that the apoptotic population resides in the upper and lower right panels of the 7-AAD/Annexin V stained cells (circled by the dotted line). PI, propidium iodide.

Further analysis of our Western blots with two apoptotic markers for MCF7 cells [39], namely caspase 9 and PARP (poly [ADP-ribose] polymerase), revealed cleavage of these proteins upon treatment with suboptimal concentrations of either AEW541 or letrozole, indicating induction of apoptosis (Figure 3b; compare lane 1 versus lanes 2 to 10). In order to further quantitate induction of apoptosis, we processed cells for flow cytometry analysis in three independent experiments. At 96 hours, cells treated with AEW541 and/or letrozole showed either no or less significant differences in their cell cycle profiles (Figure 4a and Table 3; compare percentages for G_1_, S and G_2_/M phases at 96 hours in Table 3). In contrast, the sub-G_1 _population appeared to increase when cells were treated with a combination of both compounds, which is indicative of an increasing pool of apoptotic cells. In order to confirm the presence of apoptotic cells, MCF7/Aro cells were double-stained with 7-AAD/Annexin V. Under these experimental conditions, Annexin V staining in Δ4A-treated cells was nearly absent as compared with cells that had been steroid deprived (Figure 4b and Table 3; compare 5.8% versus 23.7% of the cell population, respectively, in the ANX% column). As a single agent, letrozole exhibited no significant increase in the apoptotic population in either cell line. The same was observed for AEW541 in MCF7/Aro cells, whereas T47D/Aro cells were sensitive to IGF-IR inhibition and increased their sub-G_1 _population. More importantly, however, combining AEW541 with letrozole enhanced the apoptosis-inducing effect of both agents alone (for example, compare 11% versus 23% Annexin V staining in MCF7/Aro cells), even to the level seen in cells that were steroid-deprived (22.5% versus 23.73%), suggesting that this combination mimics the effect of depriving the cells of estrogens. Taken together, our data would suggest that a combination of AEW541 and letrozole is synergistic through increasing apoptotic responses in estrogen-dependent cells.

## Discussion

Crosstalk between the ER and IGF-IR pathways is critical for normal breast development, but also for the initiation, maintenance, and progression of breast cancer [25]. This would argue that a combined inhibition of both pathways is necessary to observe synergistic antiproliferative/proapoptotic effects in breast cancer cells. Indeed, by combining a selective IGF-IR inhibitor, AEW541 [18], with letrozole, we provide evidence for synergistic inhibition of proliferation of two independent aromatase over-expressing breast cancer cell lines, namely MCF7/Aro and T47D/Aro. These *in vitro *studies demonstrate the potential value of the combination. However, the translation of these observations into the clinic requires further studies, including assessment of the combination in appropriate animal models. Nevertheless, to our knowledge, this is the first study that clearly establishes the possibility that synergistic responses can be obtained in the clinic by combining IGF-IR inhibition with aromatase inhibition in the estrogen-dependent cancer setting.

It is worth noting that a similar rationale was previously applied in two different breast cancer xenograft models, in which combination of the anti-estrogen tamoxifen with an IGF-IR antibody proved to be more effective in suppressing tumor growth *in vivo *as compared with the single agent alone [38,40]. Further analysis of these tumor samples after 50 days of tamoxifen treatment alone revealed increased levels of IGF-IR [38]. Similar effects were observed with letrozole, for which increases in erbB2 and ERα levels were observed 4 weeks after initiating treatment [41]. We also observed in our *in vitro *models that letrozole decreases IGF-IR levels within 24 hours, followed by a clear increase in IGF-IR and ERα levels 72 hours later. The reason for this effect is not well understood. It is known that addition of an IGF-IR antibody decreases the levels of the receptor, most likely by mimicking IGF-I ligand in its ability to increase endocytosis and degradation of the receptor [42]. It therefore may be that as cells adapt to the presence of anti-endocrine therapy, the receptor is stabilized in the absence of ligand and sensitized to any ligand which becomes later available [43]. Interestingly, we observed an additive effect of AEW541 and letrozole leading to increased IGF-IRβ levels. Although this observation fits with previously published *in vivo *observations, we feel that it also has important implications for selecting a tyrosine kinase inhibitor for combination with aromatase inhibitors. More specifically, recent work indicates epidermal growth factor receptor (EGFR) signaling in breast cancer cells cannot compensate for loss of IGF-IR signaling [44]. Hence, although EGFRmay also be upregulated in response to anti-endocrine agents, a molecule that inhibits IGF-IR signaling would be predicted by these data to be more effective as an anticancer agent.

MCF7 cells over-expressing aromatase (MCF7/Aro) not only mimic *in vitro *the estrogen dependency of breast cancer, but they also have been shown to be a predictive preclinical model for anti-endocrine therapies in the clinic [37]. Although we could show that IGF-IR signaling is required for estrogen-dependent proliferation of both MCF7/Aro and, to a lesser extent, T47D/Aro cells, the fact that we did not observe a synergistic effect on cell cycle arrest when combining suboptimal concentrations of AEW541 with letrozole suggests that estrogen signaling is upstream and/or independent of IGF-IR. Both E_2 _and IGF-I synergistically provide proliferation signals to breast cancer cells, but E_2 _is essential for the proliferation of MCF7 cells [45]. Indeed, IGF-I signaling is nonmitogenic in the absence of ERα [45-47] and may even play a role in promoting invasion in ER-negative breast cancers [47]. This, however, does not mean that IGF-IR signaling is expendable in breast carcinogenesis. Transgenic mice for the IGF-I pathway, over-expressing IGF-I, IGF-IR, or IRS1 and IRS2 in the mammary gland, all develop breast adenocarcinomas [12,13,48,49]. Hence, although our data would suggest that IGF-IR is not always the primary proliferative signal in estrogen-dependent breast cancer, it may provide critical pro-survival signals.

Several data support this hypothesis. First, we observed AEW541 as a single agent induced apoptosis in T47D/Aro cells and could greatly enhance the proapoptotic potential of letrozole in MCF7/Aro cells. Second, combination of AEW541 and letrozole was not synergistic for inducing cell cycle arrest in cells, but did appear to be synergistic in MCF7/Aro cells via the decrease in levels of phospho-PKB/Akt, which is a classical target of PI3K signaling and mediator of prosurvival events. Finally, we observed that letrozole consistently sensitized cells to addition of AEW541, as reflected in higher dose response index values for AEW541. This suggests that aromatase plays a central role in mediating estradiol-dependent proliferation/survival, not only through ER but also through IGF-IR. Hence, although IGF-IR can in its own right transform cells and act synergistically with estrogen-signaling, it appears that the ability to promote survival of breast cancer cells may also be a critical function.

One of the advantages of a combination therapy is to prevent or delay the onset of resistance. Much work has been conducted on the topic of development of resistance to tamoxifen, which has an average time to progression of 6 months [50,51]. Despite the benefits of anti-endocrine therapy to breast cancer patients, a subset of breast cancers eventually develop resistance, thereby circumventing long-term deprivation of estrogen [52]. The mechanisms underlying this process have been modeled by several groups *in vitro*, in which upregulation of growth factor receptor pathways (namely Her2 or EGFR) in ER-positive cells led to a loss of ER dependency [53,54]. Notably, IGF-IR has also been linked *in vitro *to development of resistance to tamoxifen [55] and trastuzumab [56]. *In vivo *work with letrozole has revealed that erbB2 is highly induced at 4 weeks of treatment, before development of resistance [41]. Notably, no study to date has linked IGF-I signaling to development of resistance *in vivo*, beyond the observation that IGF-IR levels increase in the presence of an anti-endocrine agent (our observations and those of Ye and coworkers [38]). However, several observations suggest that IGF-IR may be required for maintaining the transformed state: IGF-IR is required for the transformation of fibroblasts by SV40 [57], it is able to improve the efficiency of EGFR signaling in tamoxifen-resistant cells [52], and its inhibition enhances the inhibitory effects of gefitinib in breast cancer cells [58]. Although letrozole has proven to be superior to tamoxifen in the treatment of endocrine-dependent breast cancers [3-6,50,51], resistance may also eventually develop [59,60].

Therefore, combined therapies of letrozole and IGF-IR inhibition may provide multiple benefits. First, inhibition of IGF-I signaling could improve the efficacy of letrozole, and is highly supported by our synergistic *in vitro *results and by other published *in vivo *studies [38,40]. Second, inhibition of both pathways could not only suppress growth of estrogen-dependent breast cancers but may also prevent development of a population of resistant cells with upregulated, IGF-IR-dependent survival pathways and invasion [19,61]. A striking example of this is a recent study revealing that inhibition of PI3K pathway signaling improves response of letrozole-insensitive xenografts [62]. It is therefore tempting to speculate on whether a delay in the onset of insensitivity would be observed if letrozole and an IGF-IR inhibitor were combined, leading to enhanced inhibition of PI3K signaling (as shown in our study). Considering that the current response time to letrozole in advanced metastatic breast cancer patients is 9.4 months (versus 6 months with tamoxifen) [50,51], a combination of an IGF-IR inhibitor with an aromatase inhibitor may prolong response time significantly. Third, it may also be possible to restore estrogen-sensitivity to a previously endocrine-insensitive tumor with an IGF-IR inhibitor. A precedent for this already exists in work conducted in letrozole-resistant xenograft models treated with gefitinib, an EGFR inhibitor [41]. Resistance is an unfortunate reality in the clinic, and a recently reported clinical study revealed that even with the combination of tamoxifen with trastuzamab, an antibody targeting EGFR, there remains a subset of patients who do not respond [63]. Hence, there is still the need for preclinical and clinical combination studies to elucidate the mechanisms of resistance developing upon anti-endocrine treatments [60].

## Conclusion

Our study emphasizes the importance of the IGF-IR pathway in the estrogen-induced proliferative response, which is in full agreement with previous studies. However, we report in this study that IGF-IR signaling plays a critical role in providing prosurvival signals in estrogen-driven proliferation of breast cancer cells. The fact that AEW541 and letrozole combine synergistically to inhibit proliferation of cells and induce apoptosis suggests that this combination therapy may improve anti-tumor efficacy in breast cancer patients. Furthermore, we provide evidence suggesting that the downregulation of the IGF-IR/PI3K/PKB/Akt pathway could be applied as predictors of efficacy in such a combination trial. We propose, therefore, that combination of letrozole and an inhibitor of IGF-IR should be explored further as a rational treatment for estrogen-dependent breast cancer.

## Abbreviations

Δ4A = androstenedione; DMSO = dimethyl sulfoxide; E_2 _= 17β-estradiol; EGFR = epidermal growth factor receptor; ER = estrogen receptor; FCS = fetal calf serum; IC_50 _= 50% inhibitory concentration; IGF = insulin-like growth factor; IGF-IR = insulin-like growth factor I receptor; IRS = insulin receptor substrate; MAPK = mitogen-activated protein kinase; MEM-EBS = minimum essential medium with Earle's balanced salts; PI3K = phosphoinositide 3-kinase; PKB = protein kinase B.

## Competing interests

JL, AP, and DBE are employees and stockholders of Novartis Pharma AG. All other authors declare that they have no competing interests.

## Authors' contributions

JL contributed to the study design, performed statistical analyses, supervised the experiments, and wrote the manuscript. AP and BC conducted the experiments with MCF7 and T47D cells, respectively. SC generated the cell lines used in the study, contributed to the study design, and supervised the experiments. DE conceived of the study and contributed to the study design. All authors read and approved the final manuscript.

## Supplementary Material

Additional file 1 A file showing synergy between AEW541 and letrozole. Specifically, the combination of AEW541 and letrozole is consistently synergistic in inhibiting androstenedione-dependent proliferation. Steroid-deprived MCF7/Aro (upper panels) and T47D/Aro (lower panels) cells were treated with Δ4A in the presence of increasing concentrations of letrozole and/or AEW541 for 6 days. Percentage inhibition of proliferation was determined as described, and combination index (CI) values of the two agents plotted, in accordance with a nonexclusive Monte Carlo extrapolation. Similarly, a conservative isobologram was plotted. The table summarizes results obtained. Fa indicates fraction affected, and DRI the dose response index. CI values above 1.1 are antagonistic, 0.9 to 1.1 are additive, 0.7 to 0.9 moderately synergistic, 0.3 to 0.7 synergistic, and under 0.3 strongly synergistic. Note that the effective dose (ED) at 50%, 75% and 90% inhibition is decreased when both agents are combined. *DRI is greater than 10 (outside of concentration range). Click here for file

Additional file 2 A file that shows that letrozole and AEW541 do not modulate p38MAPK signaling. Steroid-deprived MCF7/Aro cells were treated for 24 hours with either no Δ4A (lane 1), Δ4A in the presence of DMSO (lane 2), or Δ4A in the presence of 92 nmol/l AEW541 (lane 3), 445 nmol/l letrozole (lane 4), or both combined (lane 5). Cells were processed for Western blotting with specific antibodies for the proteins indicated. Note that samples correspond to data shown in Figure 3. Click here for file

## References

[B1] Smith IE, Dowsett M (2003). Aromatase inhibitors in breast cancer. N Engl J Med.

[B2] Simpson D, Curran MP, Perry CM (2004). Letrozole: a review of its use in postmenopausal women with breast cancer. Drugs.

[B3] Eiermann WPS, Appfelstaedt J, Llombart-Cussac A, Eremin J, Vinholes J, Mauriac L, Ellis M, Lassus M, Chaudri-Ross HA, Dugan M, Borgs M, Letrozole Neo-Adjuvant Breast Cancer Study Group (2001). Preoperative treatment of postmenopausal breast cancer patients with letrozole: A randomized double-blind multicenter study. Ann Oncol.

[B4] Ellis MJCA, Singh B, Mauriac L, Llombert-Cussac A, Jänicke F, Miller WR, Evans DB, Dugan M, Brady C, Quebe-Fehling E, Borgs M (2001). Letrozole is more effective neoadjuvant endocrine therapy than tamoxifen for ErbB-1- and/or ErbB-2-positive, estrogen receptor-positive primary breast cancer: evidence from a phase III randomized trial. J Clin Oncol.

[B5] Keshaviah A, Coates AS, Mouridsen H, Mauriac L, Forbes JF, Paridaens R, Castiglione-Gertsch M, Gelber RD, Rabaglio M, Smith I, Wardley A, Price KN, Goldhirsch A, Breast International Group (BIG) 1–98 Collaborative Group TB (2005). A comparison of letrozole and tamoxifen in postmenopausal women with early breast cancer. N Engl J Med.

[B6] Coates ASKA, Thürlimann B, Mouridsen H, Mauriac L, Forbes JF, Paridaens R, Castiglione-Gertsch M, Gelber RD, Colleoni M, Láng I, Del Mastro L, Smith I, Chirgwin J, Nogaret JM, Pienkowski T, Wardley A, Jakobsen EH, Price KN, Goldhirsch A (2007). Five years of letrozole compared with tamoxifen as initial adjuvant therapy for postmenopausal women with endocrine-responsive early breast cancer: update of study BIG 1–98. J Clin Oncol.

[B7] Goss PEIJ, Martino S, Robert NJ, Muss HB, Piccart MJ, Castiglione M, Tu D, Shepherd LE, Pritchard KI, Livingston RB, Davidson NE, Norton L, Perez EA, Abrams JS, Therasse P, Palmer MJ, Pater JL (2003). A randomized trial of letrozole in postmenopausal women after five years of tamoxifen therapy for early-stage breast cancer. N Engl J Med.

[B8] Osborne CK, Shou J, Massarweh S, Schiff R (2005). Crosstalk between estrogen receptor and growth factor receptor pathways as a cause for endocrine therapy resistance in breast cancer. Clin Cancer Res.

[B9] Foulstone E, Prince S, Zaccheo O, Burns JL, Harper J, Jacobs C, Church D, Hassan AB (2005). Insulin-like growth factor ligands, receptors, and binding proteins in cancer. J Pathol.

[B10] Mitsiades CS, Mitsiades N (2005). Treatment of hematologic malignancies and solid tumors by inhibiting IGF receptor signaling. Expert Rev Anticancer Ther.

[B11] Yu H, Rohan T (2000). Role of the insulin-like growth factor family in cancer development and progression. J Natl Cancer Inst.

[B12] Carboni JM, Lee AV, Hadsell DL, Rowley BR, Lee FY, Bol DK, Camuso AE, Gottardis M, Greer AF, Ho CP, Hurlburt W, Li A, Saulnier M, Velaparthi U, Wang C, Wen ML, Westhouse RA, Wittman M, Zimmermann K, Rupnow BA, Wong TW (2005). Tumor development by transgenic expression of a constitutively active insulin-like growth factor I receptor. Cancer Res.

[B13] Jones RACC, Gunther EJ, Chodosh LA, Petrik JJ, Khokha R, Moorehead RA (2007). Transgenic overexpression of IGF-IR disrupts mammary ductal morphogenesis and induces tumor formation. Oncogene.

[B14] Lopez T, Hanahan D (2002). Elevated levels of IGF-1 receptor convey invasive and metastatic capability in a mouse model of pancreatic islet tumorigenesis. Cancer Cell.

[B15] DiGiovanni J, Bol DK, Wilker E, Beltran L, Carbajal S, Moats S, Ramirez A, Jorcano J, Kiguchi K (2000). Constitutive expression of insulin-like growth factor-1 in epidermal basal cells of transgenic mice leads to spontaneous tumor promotion. Cancer Res.

[B16] Sachdev D, Yee D (2006). Inhibitors of insulin-like growth factor signaling: a therapeutic approach for breast cancer. J Mammary Gland Biol Neoplasia.

[B17] Wang Y, Sun Y (2002). Insulin-like growth factor receptor-1 as an anti-cancer target: blocking transformation and inducing apoptosis. Curr Cancer Drug Targets.

[B18] Garcia-Echeverria C, Pearson MA, Marti A, Meyer T, Mestan J, Zimmermann J, Gao J, Brueggen J, Capraro HG, Cozens R, Evans DB, Fabbro D, Furet P, Porta DG, Liebetanz J, Martiny-Baron G, Ruetz S, Hofmann F (2004). *In vivo *antitumor activity of NVP-AEW541-A novel, potent, and selective inhibitor of the IGF-IR kinase. Cancer Cell.

[B19] Tao YPV, Bourhis J, Deutsch E (2007). Mechanisms of disease: signaling of the insulin-like growth factor 1 receptor pathway: therapeutic perspectives in cancer. Nat Clin Pract Oncol.

[B20] Scotlandi K, Manara MC, Nicoletti G, Lollini PL, Lukas S, Benini S, Croci S, Perdichizzi S, Zambelli D, Serra M, García-Echeverría C, Hofmann F, Picci P (2005). Antitumor activity of the insulin-like growth factor-I receptor kinase inhibitor NVP-AEW541 in musculoskeletal tumors. Cancer Res.

[B21] Lee AV, Cui X, Oesterreich S (2001). Cross-talk among estrogen receptor, epidermal growth factor, and insulin-like growth factor signaling in breast cancer. Clin Cancer Res.

[B22] Hamelers IH, Steenbergh PH (2003). Interactions between estrogen and insulin-like growth factor signaling pathways in human breast tumor cells. Endocr Relat Cancer.

[B23] Dupont J, Karas M, LeRoith D (2000). The potentiation of estrogen on insulin-like growth factor I action in MCF-7 human breast cancer cells includes cell cycle components. J Biol Chem.

[B24] Lai A, Sarcevic B, Prall OW, Sutherland RL (2001). Insulin/insulin-like growth factor-I and estrogen cooperate to stimulate cyclin E-Cdk2 activation and cell Cycle progression in MCF-7 breast cancer cells through differential regulation of cyclin E and p21(WAF1/Cip1). J Biol Chem.

[B25] Thorne C, Lee AV (2003). Cross talk between estrogen receptor and IGF signaling in normal mammary gland development and breast cancer. Breast Dis.

[B26] Peyrat JP, Bonneterre J, Beuscart R, Djiane J, Demaille A (1988). Insulin-like growth factor 1 receptors in human breast cancer and their relation to estradiol and progesterone receptors. Cancer Res.

[B27] Lee AV, Hilsenbeck SG, Yee D (1998). IGF system components as prognostic markers in breast cancer. Breast Cancer Res Treat.

[B28] Lee AV, Jackson JG, Gooch JL, Hilsenbeck SG, Coronado-Heinsohn E, Osborne CK, Yee D (1999). Enhancement of insulin-like growth factor signaling in human breast cancer: estrogen regulation of insulin receptor substrate-1 expression *in vitro* and *in vivo*. Mol Endocrinol.

[B29] Pekonen F, Partanen S, Makinen T, Rutanen EM (1988). Receptors for epidermal growth factor and insulin-like growth factor I and their relation to steroid receptors in human breast cancer. Cancer Res.

[B30] Foekens JA, Portengen H, Janssen M, Klijn JG (1989). Insulin-like growth factor-1 receptors and insulin-like growth factor-1-like activity in human primary breast cancer. Cancer.

[B31] Bonneterre J, Peyrat JP, Beuscart R, Demaille A (1990). Prognostic significance of insulin-like growth factor 1 receptors in human breast cancer. Cancer Res.

[B32] Papa V, Gliozzo B, Clark GM, McGuire WL, Moore D, Fujita-Yamaguchi Y, Vigneri R, Goldfine ID, Pezzino V (1993). Insulin-like growth factor-I receptors are overexpressed and predict a low risk in human breast cancer. Cancer Res.

[B33] Hankinson SE, Willett WC, Colditz GA, Hunter DJ, Michaud DS, Deroo B, Rosner B, Speizer FE, Pollak M (1998). Circulating concentrations of insulin-like growth factor-I and risk of breast cancer. Lancet.

[B34] Sun XZ, Zhou D, Chen S (1997). Autocrine and paracrine actions of breast tumor aromatase. A three-dimensional cell culture study involving aromatase transfected MCF-7 and T-47D cells. J Steroid Biochem Mol Biol.

[B35] Chou TC, Talalay P (1984). Quantitative analysis of dose-effect relationships: the combined effects of multiple drugs or enzyme inhibitors. Adv Enzyme Regul.

[B36] Lee AV, Weng CN, Jackson JG, Yee D (1997). Activation of estrogen receptor-mediated gene transcription by IGF-I in human breast cancer cells. J Endocrinol.

[B37] Long BJ, Jelovac D, Handratta V, Thiantanawat A, MacPherson N, Ragaz J, Goloubeva OG, Brodie AM (2004). Therapeutic strategies using the aromatase inhibitor letrozole and tamoxifen in a breast cancer model. J Natl Cancer Inst.

[B38] Ye JJ, Liang SJ, Guo N, Li SL, Wu AM, Giannini S, Sachdev D, Yee D, Brunner N, Ikle D, Fujita-Yamaguchi Y (2003). Combined effects of tamoxifen and a chimeric humanized single chain antibody against the type I IGF receptor on breast tumor growth *in vivo*. Horm Metab Res.

[B39] Thiantanawat A, Long BJ, Brodie AM (2003). Signaling pathways of apoptosis activated by aromatase inhibitors and antiestrogens. Cancer Res.

[B40] Cohen BD, Baker DA, Soderstrom C, Tkalcevic G, Rossi AM, Miller PE, Tengowski MW, Wang F, Gualberto A, Beebe JS, Moyer JD (2005). Combination therapy enhances the inhibition of tumor growth with the fully human anti-type 1 insulin-like growth factor receptor monoclonal antibody CP-751,871. Clin Cancer Res.

[B41] Jelovac D, Sabnis G, Long BJ, Macedo L, Goloubeva OG, Brodie AM (2005). Activation of mitogen-activated protein kinase in xenografts and cells during prolonged treatment with aromatase inhibitor letrozole. Cancer Res.

[B42] Sachdev D, Li SL, Hartell JS, Fujita-Yamaguchi Y, Miller JS, Yee D (2003). A chimeric humanized single-chain antibody against the type I insulin-like growth factor (IGF) receptor renders breast cancer cells refractory to the mitogenic effects of IGF-I. Cancer Res.

[B43] Stewart AJ, Westley BR, May FE (1992). Modulation of the proliferative response of breast cancer cells to growth factors by oestrogen. Br J Cancer.

[B44] Riedemann J, Sohail M, Macaulay VM (2007). Dual silencing of the EGF and type 1 IGF receptors suggests dominance of IGF signaling in human breast cancer cells. Biochem Biophys Res Commun.

[B45] Burg B van der, Rutteman GR, Blankenstein MA, de Laat SW, van Zoelen EJ (1988). Mitogenic stimulation of human breast cancer cells in a growth factor-defined medium: synergistic action of insulin and estrogen. J Cell Physiol.

[B46] Oesterreich S, Zhang P, Guler RL, Sun X, Curran EM, Welshons WV, Osborne CK, Lee AV (2001). Re-expression of estrogen receptor alpha in estrogen receptor alpha-negative MCF-7 cells restores both estrogen and insulin-like growth factor-mediated signaling and growth. Cancer Res.

[B47] Surmacz E, Bartucci M (2004). Role of estrogen receptor alpha in modulating IGF-I receptor signaling and function in breast cancer. J Exp Clin Cancer Res.

[B48] Hadsell DL, Murphy KL, Bonnette SG, Reece N, Laucirica R, Rosen JM (2000). Cooperative interaction between mutant p53 and des(1–3)IGF-I accelerates mammary tumorigenesis. Oncogene.

[B49] Dearth RK, Cui X, Kim HJ, Kuiatse I, Lawrence NA, Zhang X, Divisova J, Britton OL, Mohsin S, Allred DC, Hadsell DL, Lee AV (2006). Mammary tumorigenesis and metastasis caused by overexpression of insulin receptor substrate (IRS)-1 or IRS-2. Mol Cell Biol.

[B50] Mouridsen H, Gershanovich M, Sun Y, Perez-Carrion R, Boni C, Monnier A, Apffelstaedt J, Smith R, Sleeboom HP, Janicke F, Pluzanska A, Dank M, Becquart D, Bapsy PP, Salminen E, Snyder R, Lassus M, Verbeek JA, Staffler B, Chaudri-Ross HA, Dugan M (2001). Superior efficacy of letrozole versus tamoxifen as first-line therapy for postmenopausal women with advanced breast cancer: results of a phase III study of the International Letrozole Breast Cancer Group. J Clin Oncol.

[B51] Mouridsen H, Gershanovich M, Sun Y, Perez-Carrion R, Boni C, Monnier A, Apffelstaedt J, Smith R, Sleeboom HP, Jaenicke F, Pluzanska A, Dank M, Becquart D, Bapsy PP, Salminen E, Snyder R, Chaudri-Ross H, Lang R, Wyld P, Bhatnagar A (2003). Phase III study of letrozole versus tamoxifen as first-line therapy of advanced breast cancer in postmenopausal women: analysis of survival and update of efficacy from the International Letrozole Breast Cancer Group. J Clin Oncol.

[B52] Nicholson RI, Hutcheson IR, Hiscox SE, Knowlden JM, Giles M, Barrow D, Gee JM (2005). Growth factor signalling and resistance to selective oestrogen receptor modulators and pure anti-oestrogens: the use of anti-growth factor therapies to treat or delay endocrine resistance in breast cancer. Endocr Relat Cancer.

[B53] Sabnis GJ, Jelovac D, Long B, Brodie A (2005). The role of growth factor receptor pathways in human breast cancer cells adapted to long-term estrogen deprivation. Cancer Res.

[B54] Britton DJ, Hutcheson IR, Knowlden JM, Barrow D, Giles M, McClelland RA, Gee JM, Nicholson RI (2006). Bidirectional cross talk between ERalpha and EGFR signalling pathways regulates tamoxifen-resistant growth. Breast Cancer Res Treat.

[B55] Parisot JP, Hu XF, DeLuise M, Zalcberg JR (1999). Altered expression of the IGF-1 receptor in a tamoxifen-resistant human breast cancer cell line. Br J Cancer.

[B56] Lu Y, Zi X, Zhao Y, Mascarenhas D, Pollak M (2001). Insulin-like growth factor-I receptor signaling and resistance to trastuzumab (Herceptin). J Natl Cancer Inst.

[B57] Sell C, Rubini M, Rubin R, Liu JP, Efstratiadis A, Baserga R (1993). Simian virus 40 large tumor antigen is unable to transform mouse embryonic fibroblasts lacking type 1 insulin-like growth factor receptor. Proc Natl Acad Sci USA.

[B58] Camirand A, Zakikhani M, Young F, Pollak M (2005). Inhibition of insulin-like growth factor-1 receptor signaling enhances growth-inhibitory and proapoptotic effects of gefitinib (Iressa) in human breast cancer cells. Breast Cancer Res.

[B59] Brodie A, Jelovac D, Macedo L, Sabnis G, Tilghman S, Goloubeva O (2005). Therapeutic observations in MCF-7 aromatase xenografts. Clin Cancer Res.

[B60] Leary A, Dowsett M (2006). Combination therapy with aromatase inhibitors: the next era of breast cancer treatment?. Br J Cancer.

[B61] Gee JMRJ, Gutteridge E, Ellis IO, Pinder SE, Rubini M, Nicholson RI (2005). Epidermal growth factor receptor/HER2/insulin-like growth factor receptor signalling and oestrogen receptor activity in clinical breast cancer. Endocr Relat Cancer.

[B62] Sabnis G, Goloubeva O, Jelovac D, Schayowitz A, Brodie A (2007). Inhibition of the phosphatidylinositol 3-kinase/Akt pathway improves response of long-term estrogen-deprived breast cancer xenografts to antiestrogens. Clin Cancer Res.

[B63] Marcom PK, Isaacs C, Harris L, Wong ZW, Kommarreddy A, Novielli N, Mann G, Tao Y, Ellis MJ (2007). The combination of letrozole and trastuzumab as first or second-line biological therapy produces durable responses in a subset of HER2 positive and ER positive advanced breast cancers. Breast Cancer Res Treat.

