# Supplementary material for: The aromatase inhibitor letrozole and inhibitors of insulin-like growth factor I receptor synergistically induce apoptosis in in vitro models of estrogen-dependent breast cancer
Source: Breast Cancer Res. 2008 Jul 8;10(4):R56. doi: 10.1186/bcr2113 (PMC2575527; doi:10.1186/bcr2113)
Supplement: Additional file 1 — A file showing synergy between AEW541 and letrozole. Specifically, the combination of AEW541 and letrozole is consistently synergistic in inhibiting androstenedione-dependent proliferation. Steroid-deprived MCF7/Aro (upper panels) and T47D/Aro (lower panels) cells were treated with Δ4A in the presence of increasing concentrations of letrozole and/or AEW541 for 6 days. Percentage inhibition of proliferation was determined as described, and combination index (CI) values of the two agents plotted, in accordance with a nonexclusive Monte Carlo extrapolation. Similarly, a conservative isobologram was plotted. The table summarizes results obtained. Fa indicates fraction affected, and DRI the dose response index. CI values above 1.1 are antagonistic, 0.9 to 1.1 are additive, 0.7 to 0.9 moderately synergistic, 0.3 to 0.7 synergistic, and under 0.3 strongly synergistic. Note that the effective dose (ED) at 50%, 75% and 90% inhibition is decreased when both agents are combined. *DRI is greater than 10 (outside of concentration range). [file bcr2113-S1.doc]

**Supplementary Table 1**
