# Supplementary figures and images for: The aromatase inhibitor letrozole and inhibitors of insulin-like growth factor I receptor synergistically induce apoptosis in in vitro models of estrogen-dependent breast cancer
Source: Breast Cancer Res. 2008 Jul 8;10(4):R56. doi: 10.1186/bcr2113 (PMC2575527; doi:10.1186/bcr2113)

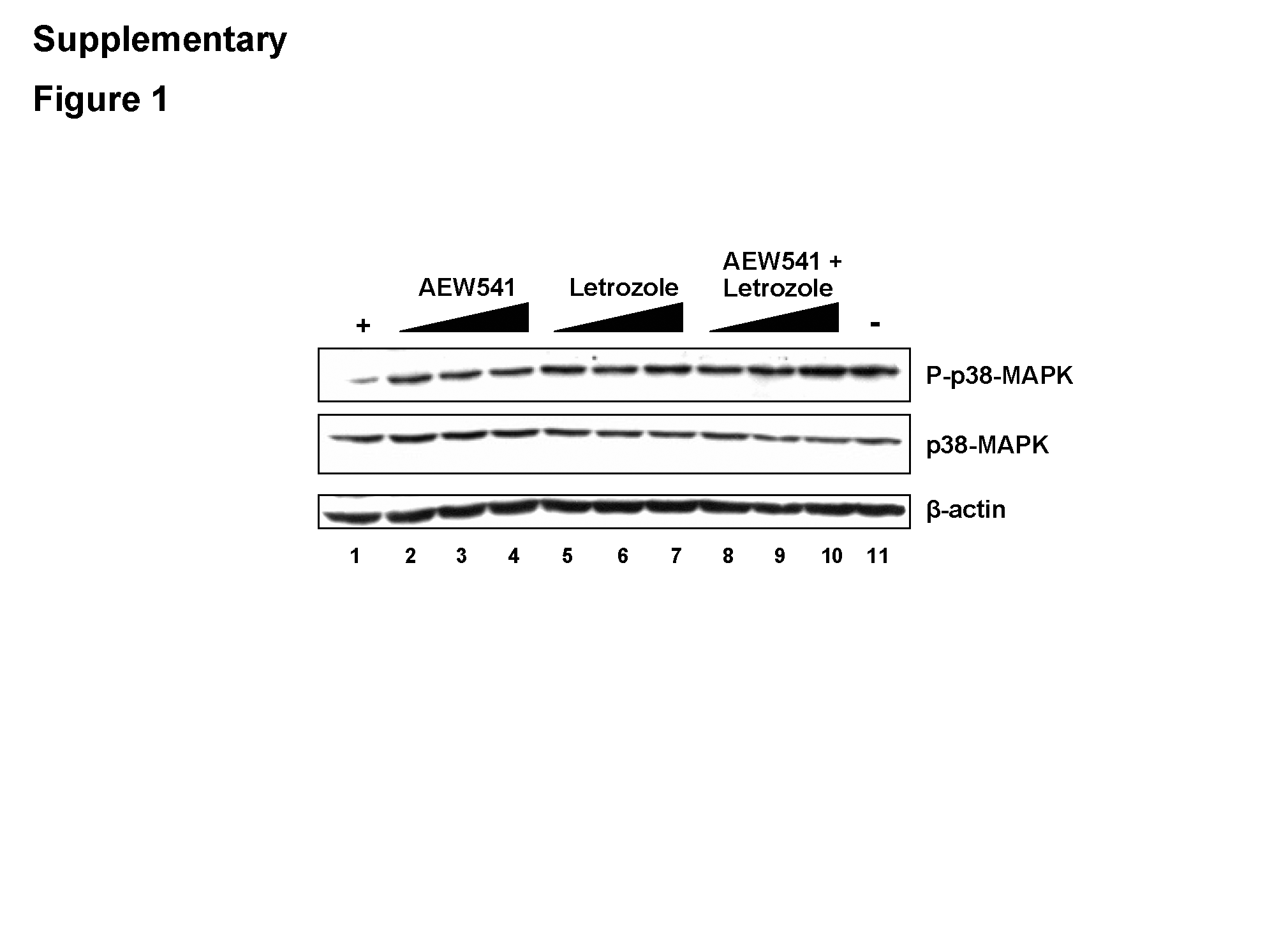

Supplement: Additional file 2 — A file that shows that letrozole and AEW541 do not modulate p38MAPK signaling. Steroid-deprived MCF7/Aro cells were treated for 24 hours with either no Δ4A (lane 1), Δ4A in the presence of DMSO (lane 2), or Δ4A in the presence of 92 nmol/l AEW541 (lane 3), 445 nmol/l letrozole (lane 4), or both combined (lane 5). Cells were processed for Western blotting with specific antibodies for the proteins indicated. Note that samples correspond to data shown in Figure 3. [file bcr2113-S2.tiff]
